# How should activity guidelines for young people be operationalised?

**DOI:** 10.1186/1479-5868-4-43

**Published:** 2007-09-21

**Authors:** Tim Olds, Kate Ridley, Melissa Wake, Kylie Hesketh, Elizabeth Waters, George Patton, Joanne Williams

**Affiliations:** 1 Nutritional Physiology Research Centre, University of South Australia, Adelaide, Australia; 2 School of Education, Flinders University, Adelaide, Australia; 3 Murdoch Children's Research Institute, Royal Children's Hospital, Melbourne, Australia; 4 Centre for Physical Activity and Nutrition Research, School ofExercise and Nutrition Sciences, Deakin University, Melbourne, Australia; 5 School of Health and Social Development, Deakin University, Melbourne, Australia

## Abstract

**Background:**

If guidelines regarding recommended activity levels for young people are to be meaningful and comparable, it should be clear how they are operationalised. It is usually open to interpretation whether young people are required to meet activity and screen time targets (1) all days of the week, (2) on most days of the week, (3) on average across all days, or (4) whether compliance should be understood as the probability that a randomly selected young person meets the guidelines on a randomly selected day. This paper studies this question using data drawn from the Australian Health of Young Victorians study.

**Methods:**

The subjects for this study were 885 13–19 year olds who recalled four days of activities using a computerised use-of-time instrument, the Multimedia Activity Recall for Children and Adolescents (MARCA). Daily minutes of moderate-to-vigorous physical activity (MVPA) and screen time were calculated. The prevalence of compliance to Australian guidelines (≥ 60 min/day of MVPA and ≤ 120 min/day of screen time outside of school hours) was calculated using the four methods.

**Results:**

The four methods resulted in significantly different prevalence estimates for compliance to the MVPA guideline (20–68%), screen guideline (12–42%) and both guidelines (2–26%). Furthermore, different individuals were identified as compliant by the different methods.

**Conclusion:**

Clarification of how compliance to guidelines should be operationalised would assist in comparisons between studies, and in consistency in determining correlates of compliance.

## Background

There has been increasing concern over the last decade at rapidly increasing levels of childhood overweight and obesity [1] and decreasing levels of fitness [2]. A number of strategies have been proposed to combat these trends, including increasing physical activity and reducing sedentary behaviour. As a result, a number of professional and government bodies around the world have issued recommendations for activity levels for young people [3,4]. These guidelines typically specify a minimum number of daily minutes of moderate-to-vigorous physical activity (MVPA), and/or a maximum number of daily minutes of exposure to television, computer and videogames (i.e. "screen time"). In late 2005, for example, the Australian Department of Health and Ageing recommended that children aged 5–18 years get a minimum of 60 minutes per day of MVPA and a maximum of 120 minutes a day of screen time for entertainment [5].

In order to monitor compliance or the extent to which populations meet guidelines, there is a need for good operationalisation of such guidelines. This would in turn allow identification at a sub-population level about whether intervention strategies are more effective for some groups than others. However, the recommendations do not always specify clearly how often children are expected to meet the guidelines, and precisely how compliance is to be calculated. Are children expected to meet the guidelines on *all *of the days sampled? While the Australian guidelines specify that children should participate in 60 minutes or more of MVPA "every day", a strict interpretation of "every day" would be quite unreasonable, not allowing for illness, travel, etc. Similarly, American guidelines [6] specify that "school-aged youth should participate *daily *in 60 minutes or more of moderate-to-vigorous physical activity" (our emphasis). There is no indication of how many days need to be sampled to determine whether the guidelines are being met. Clearly fewer children would comply with the guidelines on a sample of fourteen days than on four. Or should we understand the guidelines to mean that children should meet the targets on *most *of the days sampled? The National Association for Sport and Physical Education recommends at least 60 minutes activity "on most days of the week" [7], but 15 minutes or more of continuous physical activity "each day". Or should their minutes of accumulated MVPA and screen time be *averaged *across all the sampled days? Or should prevalence be calculated at the *child X day *level (i.e. the probability that a randomly chosen child will meet the guidelines on a randomly chosen day)? These different methods of assessing compliance to the guidelines may result in different prevalence estimates. Furthermore, if different children are identified as compliant using different methods, analysis of the factors associated with compliance, or analysis of the success of interventions, may yield differing results. Methodological consistency in estimating the prevalence of compliance would also improve comparison between studies.

The aim of this study was to compare the prevalence of compliance to Australian activity guidelines in a sample of 13–19 year olds calculated using different methods, and to quantify the extent to which the same adolescents are identified as compliant by the different methods.

## Methods

### The dataset

The subjects for this study were drawn from the third wave of a longitudinal survey conducted in Victoria, Australia. The sampling procedures and methods have been described elsewhere [8-10]. Of the original 1943 children aged 5–8 years who were recruited in 1997, 1569 children were resampled in 2000, and 885 adolescents were located and provided complete data in 2004–5. Physical activity was recalled for four days in this 2005 wave using the Multimedia Activity Recall for Children and Adolescents (MARCA) and were thus included in these analyses.

The MARCA is a computerised use-of-time instrument which allows young people to recall everything they did on the previous day from the time they woke up to the time they went to bed, choosing from over 250 activities, and using time slices as fine as five minutes. The MARCA's validity is comparable to similar multimedia and pencil-and-paper instruments [11], with correlations of rho = 0.57 and 0.41 for Physical Activity Level (PAL; average energy expenditure in METs) and daily minutes of moderate-to-vigorous physical activity (MVPA) in adolescents aged 11 and over, when compared to accelerometry. Test-retest reliability is high (rho = 0.83–0.84 for PAL, MVPA and screen time). Each activity in the MARCA is linked to a compendium which assigns it an energy cost [12]. The MARCA was usually administered in school time in small group one one-on-one interviews.

### Data analysis

Each adolescent recalled four days in the years 2005–2006, including at least one weekday and at least one weekend day. MVPA was defined as any activity requiring at least 3 METs [13]. The four days of data collection were spread throughout the school year. The total number of minutes devoted to such activities was calculated for each recall day. Screen time included out-of-school television/video/DVD, computer use and electronic games.

Four methods of calculating prevalence were compared:

(1) The *All Days *method. Adolescents were considered compliant if they met the guidelines on all of the 4 days.

(2) The *Most Days *method. Adolescents were considered compliant if they met the guidelines on at least 3 of the 4 days.

(3) The *Four-Day Average *method. Adolescents were considered compliant to the MVPA guidelines if their MVPA, averaged over four days, was ≥ 60 minutes a day; compliant to the screen guidelines if their average daily screen time was ≤ 120 minutes a day; and compliant to both guidelines if both thresholds were met.

(4) The *Child X Day *method. Prevalence was calculated as the probability that a randomly chosen adolescent on a randomly chosen day would meet the guidelines. To calculate this prevalence estimate, it is simply a matter of calculating the proportion of all reported days which meet the guideline.

Prevalence was calculated separately for MVPA, screen time, and both conjointly.

In addition to exploring differences in prevalence estimates, a second aim of this study was to determine whether the same children were identified as compliant or non-compliant by the different methods. This could only be done for the All Days, Most Days and Four-Day Average methods, because only these used the child (as opposed to the child-day) as the analytical unit. Cochran's Q was used to test for differences in the proportions between the All Days, Most Days and Four-Day Average methods, and McNemar's tests were used in post-hoc pairwise comparisons.

## Results

### Subject characteristics

The subject characteristics are shown in Table 1.

**Table 1:** Subject Characteristics

	*Boys *(n = 371)	*Girls *(n = 400)	*All *(n = 771)
*Age (years)*	16.1 (1.3)	16.0 (1.2)	16.0 (1.2)
*Height (cm)*	174.3 (8.2)	164.3 (6.8)	169.0 (9.0)
*Mass (kg)*	68.7 (14.4)	60.8 (11.5)	64.5 (13.6)
*MVPA (min/day)*	134 (87)	78 (55)	105 (77)
*Screen time (min/day)*	203 (106)	147 (84)	174 (99)

Data are shown as mean (SD). The values shown for MVPA and screen time are four-day averages. MVPA = moderate-to-vigorous physical activity.

### Prevalence estimates

About 60% of the days recalled were non-school days, reasonably reflecting a typical year for Australians of this age when weekends, holidays, sick days and teacher-free days are taken into account, and considering that not all young people of this age are at school. The weekdays sampled favoured Mondays and Tuesdays (15% and 12% respectively) above Wednesdays, Thursdays and Fridays (8%, 6% and 9%). However, more of the recalled days were in winter (38%) and autumn (30%), than in spring (24%) and summer (8%), because data collection was limited during the summer holidays. As a result, holidays were also under-represented. Prevalence estimates derived using the four methods are shown in Table 2.

**Table 2:** Prevalence (%) and 95% confidence interval (in parentheses) of compliance with MVPA, screen time and both guidelines, calculated using the four methods

*Method*	*% meeting MVPA guideline*	*% meeting screen guideline*	*% meeting both guidelines*
*All Days*	20 (17–23)	12 (10–14)	2 (1–3)
*Most Days*	46 (42–50)	29 (26–32)	12 (10–14)
*Four-Day Average*	68 (65–71)	34 (31–37)	26 (23–29)
*Child X Day*	57 (54–60)	42 (39–45)	26 (23–29)

**Figure 1:**
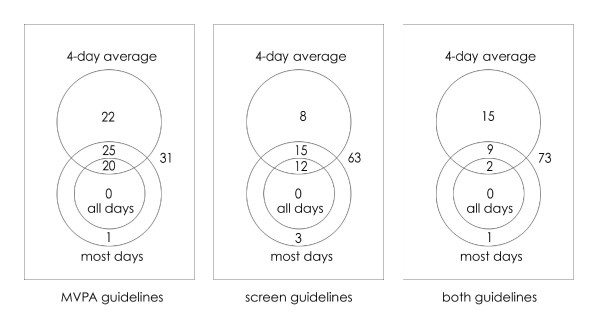
Venn diagrams showing the percentage of children defined as compliant or non-compliant by the *Four-Day Average *(top circle), *Most Days *(larger lower circle), and *All Days *methods (smaller lower circle). The left panel shows results for the MVPA guidelines, the central panel for screen time guidelines, and the right panel for both sets of guidelines conjointly. For example, in the leftmost panel, 22% of children are classified as compliant only by the Four-day Average method; 1% as compliant only by the Most Days method; 20% by both the Four-day Average method and the All Days method; 25% by the Four-day Average and Most Days methods, but not by the All Days method; while 31% are classified as not compliant by any method.

Figure 1 illustrates the agreement between children identified as compliant by the All Days, Most Days and Four-Day Average methods (i.e. the three methods where the child, as opposed to the child X day, is the unit of analysis). For MVPA, 45% of children were classified as compliant by both the Four-Day Average and Most Days methods, and 23% by only one of those methods. For screen time, 27% of children were classified as compliant by both methods, and 11% by only one of the methods. For both guidelines, 11% of children were classified as compliant by both methods, and 16% by only one of the methods.

Cochran's Q showed significant differences in the proportions of children classified as compliant by the three methods for MVPA (χ^2 ^= 628.5, *P *< 0.0001), screen time (χ^2 ^= 284.3, *P *< 0.0001), and both guidelines (χ^2 ^= 275.5, *P *< 0.0001). Using McNemar's test, there were significant differences between the categorisation of children by the Four-Day Average and Most Days methods for MVPA (χ^2 ^= 185.6, *P *< 0.0001), screen time (χ^2 ^= 36.4, *P *< 0.0001) and both guidelines (χ^2 ^= 73.0, *P *< 0.0001). There were also significant differences between the categorisation of children by the Four-Day Average and All Days methods for MVPA (χ^2 ^= 401.2, *P *< 0.0001), screen time (χ^2 ^= 196.0, *P *< 0.0001) and both guidelines (χ^2 ^= 191.0, *P *< 0.0001). Finally, there were significant differences between the categorisation of children by the Most Days and All Days methods for MVPA (χ^2 ^= 227.0, *P *< 0.0001), screen time (χ^2 ^= 135.0, *P *< 0.0001) and both guidelines (χ^2 ^= 100.0, *P *< 0.0001).

## Discussion

### Comparison of the methods

The main finding of this study was that estimates of the prevalence of compliance to activity guidelines varied according to how the guidelines were interpreted. Prevalence estimates ranged from 20 to 68% for MVPA, 12 to 42% for screen time, and 2 to 26% for both guidelines. Furthermore, children were classified significantly differently by the different methods. In particular, only a very small proportion of children were compliant using the All Days method. The results from studies using different methods would therefore not be comparable, and predictors and correlates of compliance calculated using one method may no longer be significant when compliance is calculated by another method.

Each of the four methods of calculating prevalence has an inherent logic and specific advantages and disadvantages. The Four-Day Average method allows data to be gathered with a single administration of a questionnaire where children are invited to recall a "typical" week. However, it is perhaps important for health that children be regularly active rather than accumulate MVPA on one or two days, because many of the benefits of physical activity may have a relatively short half-life. On the other hand, one day of very high screen time may carry the four-day average over the threshold, but there is evidence that prolonged sedentary periods may be more harmful than the same amount of time accumulated in shorter bouts [14]. Furthermore, using the Four-Day Average method does not allow researchers to explore associations between characteristics of the day (e.g. weather conditions, school vs non-school) and whether the guidelines have been met.

The Most Days method is simple and easily comprehensible, although it is not made explicit in the guidelines. It would allow for the occasional days where children could not be expected to meet the guidelines, through sickness or travel for example. However, if one-day recalls are used, monitoring compliance may require multiple administrations of an instrument with consequent increases in respondent burden. If multi-day recalls are used at a single administration of a questionnaire, validity has been shown to decrease [11].

**Figure 2:**
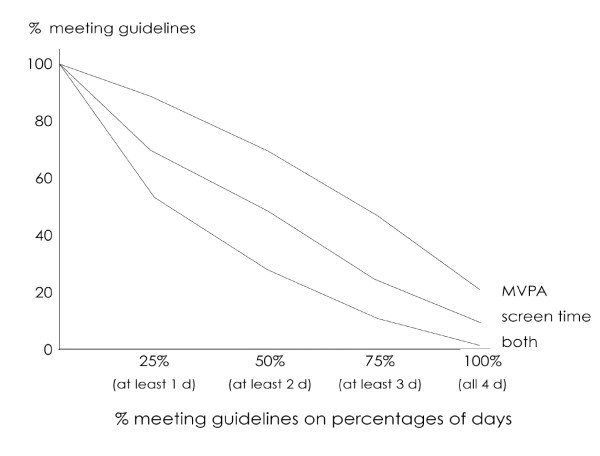
The relationship between the number of days on which young people meet the guidelines and the percentage of young people meeting those guidelines on at least that number of days. For example, about 30% of young people meet both guidelines on at least 50% of days. Data are shown for MVPA guidelines (top line), screen time guidelines (middle line), and both guidelines (bottom line).

A further complication when using the Most Days and All Days criteria is that the percentages of young people classified as compliant are likely to vary with the number of days being recalled. If seven days are recalled, for example, "most days" (i.e. four days) constitutes only 57% of days. If four days are recalled, "most days" (i.e. three days) constitutes 75% of all days. One would expect overall compliance to be lower if young people were required to meet the guidelines on a larger percentage of days. Figure 2 shows the percentage of young people meeting the guidelines on at least 1, 2, 3 and 4 days (i.e. 25%, 50%, 75% and 100% of days).

Based on these relationships, we would expect that on at least half of all days, about 70% of young people would comply with the MVPA guidelines, about 50% with the screen time guidelines, and about 30% with both guidelines. Plotting curves of this sort allow estimates of compliance rates with various percentages of days to be calculated, even if different numbers of days are sampled in different surveys.

The Child X Day method, in the absence of clearer specifications, is a logical way of interpreting the guidelines – the probability that a randomly chosen child on a randomly chosen day will meet the guidelines. This way of construing the prevalence issue, using the child-day rather than the child as the unit of analysis, is at first sight unusual, but has the advantage of making possible an analysis of the relationship between characteristics of the day (e.g. weather conditions, school/weekend/holiday) and compliance. One disadvantage of this method is that, while it is simple to operationalise from a monitoring point of view, it is less intuitive for young people, parents and care-givers.

### Strengths and limitations

Strengths of this study include the large sample size, its community-based nature, the reliability and detailed nature of the use-of-time data achieved by the computerised MARCA, and the novelty of the question raised. Limitations include the attrition rate since the study commenced in 1997, with the sample unlikely to now be representative of the whole population. For example, those who have remained in the study might exhibit greater organisation, diligence or family support, which are likely to be associated with higher levels of MVPA and lower levels of screen time. While this precludes reliable reporting of population prevalence and may limit generalisability, it should not invalidate associations between variables such as are the focus of this report. The days recalled in this study contained a representative mix of school and non-school days. However, because the instrument was usually administered in school, summer days and holidays were under-represented. It is possible that higher levels of screen time are accumulated on holidays, so the estimates of the prevalence of compliance here may be artificially high.

### Estimates of population compliance

The main thrust of this paper is to highlight the different results obtained when different operationalisations of activity guidelines are used. Not withstanding the limitations noted, the data also provide an insight into the extent of compliance with the Australian guidelines. Whatever method was used, the prevalence of compliance was low. At most about a quarter of the adolescents in this sample met both guidelines, and less than half the screen guidelines. Only 1% of adolescents met both guidelines on all four days. In general, young people were about twice as likely to meet the MVPA guidelines (20–68%) as the screen time guidelines (12–42%).

### Future recommendations

In much the same way as internationally-agreed BMI cutpoints represented a major advance without which secular trend and cross-country comparisons were impossible, this paper highlights how important consistency is when operationalising physical activity guidelines for youth. The choice of method will perforce be imperfect, but should nonetheless be agreed for these purposes. Future research must address the physical activity and inactivity criteria most closely associated with health and illness.

## Authors' contributions

TO conceived the idea for the paper, is co-author of the MARCA, and was a Chief Investigator on the HOYVS project. KR is co-author of the MARCA and assisted with analysing MARCA data. MW helped refine the conceptual framework and was team leader on the HOYVS project. KH, GP, EW and JW contributed to the conceptual framework and were Chief Investigators on the HOYVS project. All authors read and approved the final manuscript.
